# Ultrasonic aspiration in neurosurgery: comparative analysis of complications and outcome for three commonly used models

**DOI:** 10.1007/s00701-019-04021-0

**Published:** 2019-08-03

**Authors:** Stephanie Henzi, Niklaus Krayenbühl, Oliver Bozinov, Luca Regli, Martin N. Stienen

**Affiliations:** 1 Department of Neurosurgery, University Hospital Zurich, Frauenklinikstrasse 10, 8091 Zurich, Switzerland; 2 Clinical Neuroscience Center, University of Zurich, Frauenklinikstrasse 10, 8091 Zurich, Switzerland

**Keywords:** Ultrasonic aspiration, Intracranial tumor, Complications, Outcome, Morbidity, Mortality

## Abstract

**Introduction:**

Ultrasonic aspiration (UA) devices are commonly used for resecting intracranial tumors, as they allow for internal debulking of large tumors, hereby avoiding damage to adjacent brain tissue during the dissection. Little is known about their comparative safety profiles.

**Methods and materials:**

We analyzed data from a prospective patient registry. Procedures using one of the following UA models were included: Integra® CUSA, Söring®, and Stryker® Sonopet. The primary endpoint was morbidity at discharge, defined as significant worsening on the Karnofsky Performance Scale. Secondary endpoints included morbidity and mortality until 3 months postoperative (M3), occurrence, type, and etiology of complications.

**Results:**

Of *n* = 1028 procedures, the CUSA was used in *n* = 354 (34.4 %), the Söring in *n* = 461 (44.8 %), and the Sonopet in *n* = 213 (20.7 %). There was some heterogeneity of study groups. In multivariable analysis, patients in the Söring (adjusted odds ratio (aOR) 1.29; 95 % confidence interval (CI), 0.80–2.08; *p* = 0.299), and Sonopet group (aOR, 0.86; 95 % CI, 0.46–1.61; *p* = 0.645) were as likely as patients in the CUSA group to experience discharge morbidity. At M3, patients in the Söring (aOR, 1.20; 95 % CI, 0.78–1.86; *p* = 0.415) and Sonopet group (aOR, 0.53; 95 % CI, 0.26–1.08; *p* = 0.080) were as likely as patients in the CUSA group to experience morbidity. There were also no differences for M3 morbidity in subgroup analyses for gliomas, meningiomas, and metastases. The grade (*p* = 0.608) and etiology (*p* = 0.849) of postoperative complications were similar.

**Conclusions:**

Neurosurgeons select UA types with regard to certain case-specific characteristics. The safety profiles of three commonly used UA types appear mostly similar.

**Electronic supplementary material:**

The online version of this article (10.1007/s00701-019-04021-0) contains supplementary material, which is available to authorized users.

## Introduction

In the past few decades, ultrasonic aspiration (UA) has become a commonly used technique in neurosurgery to help treat a variety of intracranial and intraspinal tumors. The main advantages of UA are that they allow for less invasive surgeries by safely debulking large tumors internally, hereby avoiding damage to adjacent brain tissue during the dissection. The ultrasonic transducer limits the damage to blood vessels and nerve fibers during tumor resection due to tissue selection, which is beneficial to the patient’s prognosis [9, 14, 17]. Additionally, it also reduces surgery time, decreases blood loss, and improves the overall quality of the operation [3, 4].

Thus, UA devices appear to be very useful tools in neurosurgery, especially when it comes to tumors, which are difficult to resect for their deep or eloquent location. More recently, UA devices have even been implemented into neuroendoscopy and may help surgeons achieve better outcomes in patients with intraventricular tumors [5, 13, 16]. Currently, there are various kinds of UAs, all produced by different companies. At our department, neurosurgeons may choose between three models, the CUSA Excel®/Clarity® (Integra®, Plainsboro, NJ (USA)), Sonopet® (Stryker®, Kalamazoo, MI (USA)), or Söring® (Söring GmbH, Quickborn, Germany), on a case-by-case basis. Whether or not any of those three UA systems provides benefits in terms of patient outcome, reduction of complications, or extend of resection (EOR) remains poorly understood.

The primary aim of this work was to compare clinical outcomes and complications of patients with intracranial tumors, resected with the help of the aforementioned three UA types.

## Materials and methods

### Study design and database

This was a retrospective cross-sectional cohort study, which used prospectively acquired patient data from the institutional database of the Department of Neurosurgery, University Hospital Zurich (USZ) [15].

### Patient identification

**Fig. 1:**
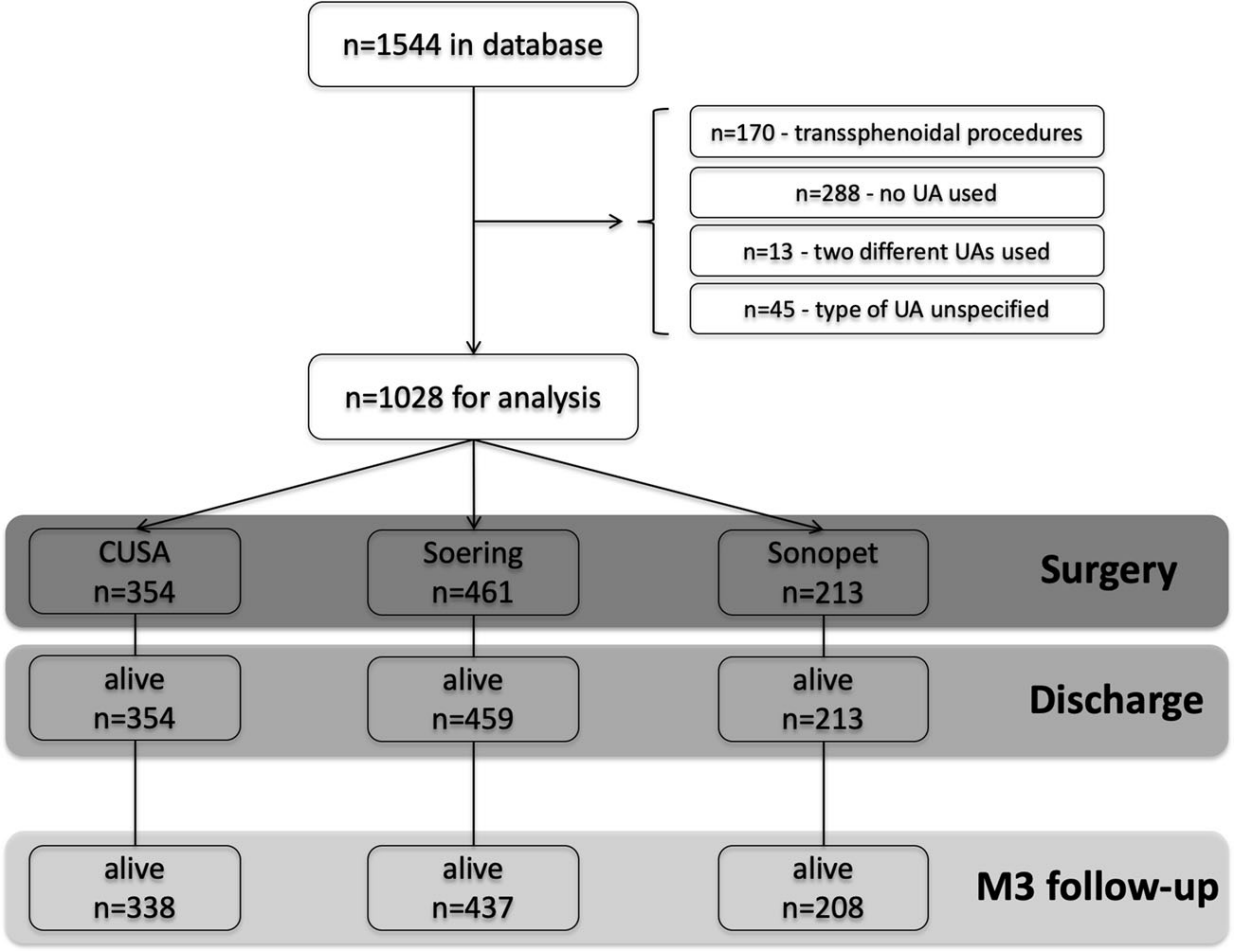
Algorithm describing how the study arrived at the final cohort size at baseline and follow-up 3 months postoperative (M3)

All patients who underwent surgery at USZ between 01/2013 and 12/2017 were considered. Patients undergoing transsphenoidal procedures were excluded (in our hands UAs are rarely used during these procedures), as well as operations in which no UA device was used. Additionally, we omitted all surgeries during which two different UA types were used or where there was no specification as to which UA was used (Fig. 1).

### Data collection

Patient’s baseline characteristics including age, sex, American Society of Anesthesiology (ASA) grading scale, smoking status, previous surgery, type of tumor, tumor location, tumor size, and functional status at hospital admission—as described by the Karnofsky Performance Scale (KPS), the modified Rankin scale (mRS), and the National Institute of Health stroke scale (NIHISS)—were extracted from the database [9]. Missing data was added by retrospective chart review, if present. The Milan Complexity Score (MCS) as defined by Ferroli et al. [8] was generated from available variables in the database and procedural complexity was stratified into low (0–2), moderate (3–5), and high (6–8). We grouped patients by their functional performance status (KPS) into good (80–100), moderate (50–70), and poor (40–0). Correspondingly, patients were categorized by the modified Rankin Scale (mRS) into good (0–1), moderate (2–3), and poor (4–5) disability, and by the National Institute of Health Stroke Scale (NIHSS) into good (0–1), moderate (2–5), and poor (≥ 6) neurological status. Furthermore, the surgeon’s level of experience was included and divided into three groups: supervised resident, attending, and senior attending.

Information regarding the type of used UA was added by reviewing electronic patient records, specifically the surgery documentation: a detailed report, which is filled out by the scrub nurse and circulator during each operation. In case the surgery documentation did not mention the use of an UA, the surgeon’s written operative report was reviewed to ensure all surgeries with UAs were correctly identified.

There were three models of UAs used at our clinic: CUSA Excel®, Sonopet®, and Söring. CUSA Excel® has recently been replaced by a newer model, CUSA Clarity®, but as both models combine common technical features and for reasons of simplicity no differentiation was made between the two.

The main outcome variable was the KPS at discharge and at M3; morbidity and mortality were constructed from the KPS data. In addition, rate, grading, and etiology of in-hospital complications, according to the Clavien-Dindo grading scale (CDG) [6, 7], were analyzed. Length of hospitalization, length of surgery, and discharge location were considered as surrogate markers for the technical success of the surgery and outcome. To determine the extent of resection (EOR), the written neuroradiology reports of postoperative MRI were reviewed. EOR was coded as either gross total resection (GTR; i.e., no residual tumor), subtotal resection (STR; i.e., residual tumor of any size), unclear (e.g., MRI conducted > 72 h after the operation or neuroradiologist unsure concerning the presence of residual tumor), or no immediate postoperative MR-imaging available.

### Statistical consideration and endpoints

Baseline characteristics were described using mean and standard deviation (SD) for interval variables and frequency and percentage for categorical variables, respectively. Imbalances between the dependent and independent groups were tested using Pearson *χ*^2^ tests, Student’s *t* tests, and uni- and multivariable analysis of variance (ANOVA and MANOVA).

The primary endpoint was morbidity at time of discharge, defined as significant worsening on the KPS. The latter was defined as a decrease of ≥ 20 points if baseline KPS ≥ 80 or as a decrease of ≥ 10 points if baseline KPS < 80. This approach has been used previously to account for the ceiling effect inherent to the KPS scale, where 10-point worsening on the lower KPS has more impact on a patient that 10-point worsening on the upper KPS [12, 18]. From here, logistic regression analysis was applied to estimate the effect size of the relationship between the UA type and the outcome of interest. First, a univariable analysis was performed to study direct relationships, expressed as odds ratios (OR) and 95 % confidence intervals (CI). A multivariable analysis was adjusted for characteristics that were found to differ at baseline, as shown in Table 1. As there were important group differences in terms of histopathological tumor types, we conducted subgroup analyses for the three most common tumor types (gliomas, metastasis, meningiomas) to account for possible confounding despite statistical adjustment.

**Table 1:** Baseline table describing patient characteristics

	CUSA	Söring	Sonopet	*p* value
Age (in years)	54.0 (15.5)	55.9 (17.5)	53.9 (15.6)	0.182
Sex				< 0.001
Male	189 (53.4 %)	198 (42.9 %)	142 (66.7 %)	
Female	165 (46.6 %)	263 (57.1 %)	71 (33.3 %)	
ASA class				< 0.001
1	34 (9.6 %)	35 (7.6 %)	32 (15.0 %)	
2	208 (58.8 %)	236 (51.2 %)	129 (60.6 %)	
3	107 (30.2 %)	181 (39.3 %)	47 (22.1 %)	
4	5 (1.4 %)	9 (1.9 %)	5 (2.3 %)	
Smoking status				0.574
Nonsmoker	209 (59.0 %)	260 (56.4 %)	134 (62.9 %)	
Active smoker	80 (22.6 %)	117 (25.4 %)	45 (21.1 %)	
Former smoker	65 (18.4 %)	84 (18.2 %)	34 (16.0 %)	
Previous surgery				0.276
Yes	66 (18.6 %)	104 (22.6 %)	39 (18.3 %)	
No	288 (81.4 %)	357 (77.4%)	174 (81.7 %)	
Type of tumor				< 0.001
Glioma	130 (36.7 %)	247 (53.6 %)	42 (19.7 %)	
Meningioma	105 (29.7 %)	49 (10.6 %)	126 (59.1 %)	
Metastasis	55 (15.5 %)	137 (29.7 %)	18 (8.5 %)	
Other	64 (18.1 %)	28 (6.1 %)	27 (12.7 %)	
Tumor location				< 0.001
Intraaxial	213 (60.2 %)	376 (81.6 %)	98 (46.0 %)	
Extraaxial	140 (39.5 %)	82 (17.8 %)	115 (54.0 %)	
Unspecified	1 (0.3 %)	3 (0.6 %)	– (0.0 %)	
Maximum tumor size (in cm)	3.8 (1.7)	3.9 (1.6)	4.1 (1.6)	0.081
Milan Complexity Score				< 0.001
Low (0–2)	135 (38.2 %)	206 (44.7 %)	78 (36.6 %)	
Moderate (3–5)	146 (41.2 %)	227 (49.2 %)	84 (39.5 %)	
High (6–8)	73 (20.6 %)	28 (6.1 %)	51 (23.9 %)	
Admission KPS				0.917
Good (80–100)	261 (73.7 %)	340 (73.8 %)	158 (74.2 %)	
Moderate (50–70)	85 (24.0 %)	106 (23.0 %)	48 (22.5 %)	
Poor (40–0)	8 (2.3 %)	15 (3.2 %)	7 (3.3 %)	
Admission mRS				0.599
Good (0–1)	202 (57.1 %)	240 (52.1 %)	119 (55.9 %)	
Moderate (2–3)	136 (38.4 %)	194 (42.1 %)	85 (39.9 %)	
Poor (4–5)	16 (4.5 %)	27 (5.8 %)	9 (4.2 %)	
Admission NIHSS				0.615
Good (0–1)	234 (66.1 %)	302 (65.5 %)	139 (65.3 %)	
Moderate (2–5)	97 (27.4 %)	136 (29.5 %)	66 (31.0 %)	
Poor (≥ 6)	23 (6.5 %)	23 (5.0 %)	8 (3.7 %)	
Level of experience				< 0.001
Supervised resident	48 (13.6 %)	89 (19.3 %)	15 (7.04 %)	
Attending	118 (33.3 %)	183 (39.7 %)	52 (24.4 %)	
Senior attending	188 (53.11 %)	189 (41.0 %)	146 (68.5 %)	
	*n* = *354* (*100* %)	*n* = *461* (*100* %)	*n* = *213* (*100* %)	

Data is presented in mean (standard deviation) or count (percent)

Italic entries were the count of the total cohort size

Secondary endpoints included morbidity and mortality (KPS, 0) at M3, occurrence, grading, and etiology of in-hospital complications, with special emphasis placed on major complications (CDG 3b−5), as well as on complications labeled as “traumatic” (= resulting from surgical trauma). We further considered the discharge location, length of surgery (LOS), length of hospitalization (LOH), and extent of resection.

Knowing that about 10 % of patients experienced the primary endpoint, we calculated that a sample of *n* = 401 patients was required in order to detect a 5 % difference in the primary endpoint with a power of 0.80 and alpha set at 0.05.

All statistical analyses were performed with Stata Version 14.2 for Mac (College Station, TX; StataCorp LLC). *P* values < 0.05 were considered as statistically significant.

### Ethical considerations

The use and workup of registry data were approved by the institutional review board. The patient’s informed consent was waived. The study protocol was approved by the local ethics committee (Kantonale Ethikkommission KEK-ZH 2012-0244) and registered at http://clinicaltrials.gov (NCT01628406).

This project was financed by the Department of Neurosurgery, USZ. It was financially supported by Stryker European Operations B.V. (Amsterdam, The Netherlands). The external funding source was not involved and did not influence data collection, measurements, interpretation, or drafting of this article.

## Results

### Patient characteristics

Out of *n* = 1544 cases in the database, 516 procedures were omitted from further analysis for the following reasons: *n* = 170 (transsphenoidal procedure), *n* = 288 (transcranial procedure, but no UA used), *n* = 13 (two different UAs used in the same procedure), and *n* = 45 (type of UA not specified) (Fig. 1). As a result, *n* = 1028 procedures were considered for final analysis, for which the CUSA was used in *n* = 354 (34.4 %), the Söring in *n* = 461 (44.8 %), and the Sonopet in *n* = 213 (20.7 %).

The baseline characteristics of all three groups are listed in Table 1. The study groups were balanced for most variables, including age, smoking status, repeated surgery, tumor size, and baseline functional status (admission KPS, mRS, and NIHSS). There were significant differences in terms of sex, ASA class, tumor location, case complexity, and level of experience. The most frequent histopathological tumor types in the CUSA group were gliomas (36.7 %), followed by meningiomas (29.7 %), and metastases (15.5 %), while in the Söring group gliomas (53.6 %) were followed by metastases (29.7 %) and meningiomas (10.6 %), and in the Sonopet group meningiomas (59.1 %) were more common than both gliomas (19.7 %) and metastases (8.5 %; *p* < 0.001).

### Primary endpoint: morbidity at discharge

Morbidity at discharge occurred in a total of *n* = 107 cases (10.4 %); 34 (9.6 %), 56 (12.1 %), and 17 (8.0 %) in the CUSA, Söring, and Sonopet group, respectively. In univariable analysis, patients in the Söring group were as likely as patients in the CUSA group to experience discharge morbidity (OR, 1.30; 95 % CI, 0.82–2.04; *p* = 0.252) and the same applied to the Sonopet group (OR, 0.81; 95 % CI, 0.44–1.50; *p* = 0.513). In multivariable analysis, adjusted for baseline differences in sex, ASA grade, type of tumor, location of tumor, case complexity (MCS), and level of experience, patients in both the Söring (aOR, 1.29; 95 % CI, 0.80–2.08; *p* = 0.299) and the Sonopet group (aOR, 0.86; 95 % CI, 0.46–1.61,; *p* = 0.645) were as likely as those in the CUSA group to experience morbidity at time of hospital discharge (Table 2).

**Table 2:** Relationship between UA type and discharge morbidity

Discharge morbidity	Univariate analysis			Multivariate analysis		
	OR	95 % CI	*p* value	OR	95% CI	*p* value
UA type*						
Söring	1.30	0.82–2.04	0.252	1.29	0.80–2.08	0.299
Sonopet	0.81	0.44–1.50	0.513	0.86	0.46–1.61	0.645
Female sex	1.40	0.94–2.10	0.101	1.37	0.91–2.09	0.135
ASA grade (per 1-step increase)	1.54	1.13–2.09	0.006	1.50	1.09–2.07	0.012
Tumor type	1.00	0.82–1.21	0.999	0.97	0.80–1.18	0.777
Extraaxial tumor location	0.65	0.41–1.02	0.060	0.78	0.49–1.25	0.304
MCS grade (per increase in category)	1.60	1.21–2.12	0.001	1.76	1.29–2.41	< 0.001
Level of experience	1.05	0.79–1.39	0.737	1.04	0.76–1.41	0.825

Uni- and multivariate logistic regression analysis estimating the relationship between UA type and morbidity at time of discharge. The multivariate analysis is adjusted for baseline differences in sex, ASA grading scale, type of tumor, location of tumor, case complexity (MCS), and level of experience. *The analysis compares the results of each listed UA type with the CUSA ultrasonic aspirator

### Secondary endpoint: morbidity at M3

Morbidity at M3 occurred in a total of *n* = 125 cases (12.2 %); 40 (11.3 %), 74 (16.1 %), and 11 (5.2 %) in the CUSA, Söring, and Sonopet group, respectively. In univariable analysis, patients in the Söring group tended to be more likely than patients in the CUSA group to experience morbidity at M3 (OR, 1.50; 95 % CI, 0.99–2.27; *p* = 0.054), but after adjusting for potential confounders the effect diminished and lost statistical significance (aOR, 1.20; 95 % CI, 0.78–1.86; *p* = 0.415). Patients in the Sonopet group were less likely than patients in the CUSA group to experience morbidity in univariable analysis (OR, 0.43; 95 % CI, 0.21–0.85; *p* = 0.016). In multivariable analysis, the effect size was slightly attenuated, with the *p* value now being slightly above the predefined cut-off for significance (aOR, 0.53; 95 % CI, 0.26–1.08; *p* = 0.080; Table 3).

**Table 3:** Relationship between UA type and morbidity at M3 follow-up

M3 morbidity	Univariate analysis			Multivariate analysis		
	OR	95 % CI	*p* value	OR	95 % CI	*p* value
UA type*						
Söring	1.50	0.99–2.27	0.054	1.20	0.78–1.86	0.415
Sonopet	0.43	0.21–0.85	0.016	0.53	0.26–1.08	0.080
Female sex	1.57	1.08–2.30	0.019	1.38	0.93–2.04	0.107
ASA grade (per 1-step increase)	2.34	1.73–3.15	< 0.001	2.06	1.51–2.82	< 0.001
Tumor type	0.94	0.78–1.13	0.488	0.95	0.79–1.15	0.696
Extraaxial tumor location	0.49	0.31–0.77	0.002	0.67	0.41–1.07	0.122
MCS grade (per increase in category)	0.97	0.74–1.27	0.834	1.16	0.85–1.57	0.646
Level of experience	0.64	0.50–0.82	< 0.001	0.74	0.57–0.97	0.031

Uni- and multivariate logistic regression analysis estimating the relationship between UA type and morbidity at time of M3 follow-up. The multivariate analysis is adjusted for baseline differences in sex, ASA grading scale, type of tumor, location of tumor, the case complexity (MCS), and level of experience. *The analysis compares the results of each listed UA type with the CUSA ultrasonic aspirator

Subgroup analyses were performed for the three major histopathological tumor types: gliomas (*n* = 419), meningiomas (*n* = 280), and metastases (*n* = 210; Supplementary tables 1–3). In multivariable analysis, glioma patients both in the Söring (aOR, 1.16; *p* = 0.630) and in the Sonopet group (aOR, 0.95; *p* = 0.935) were as likely to experience M3 morbidity as glioma patients in the CUSA group. Similarly, meningioma patients both in the Söring (aOR, 0.87; *p* = 0.903) and in the Sonopet group (aOR, 0.25; *p* = 0.100) were as likely to experience morbidity at M3 as meningioma patients in the CUSA group. Lastly, metastasis patients both in the Söring (aOR, 0.76; *p* = 0.469) and in the Sonopet group (aOR, 0.57; *p* = 0.432) were as likely to experience morbidity at M3 as metastasis patients in the CUSA group.

### Secondary endpoints: mortality

In-hospital mortality was *n* = 2 in the series (0.19 %), both of which occurred in the Söring group (*p* = 0.292).

Mortality at M3 occurred in *n* = 45 patients (4.4 %); 16 (4.5 %), 24 (5.2 %), and 5 (2.3 %) in the CUSA, Söring, and Sonopet group, respectively. In univariable analysis, patients in both the Söring (OR, 1.16; 95 % CI, 0.61–2.22; *p* = 0.653) and the Sonopet group (OR, 0.51, 95 % CI, 0.18–1.41; *p* = 0.192) were as likely as patients in the CUSA group to experience mortality at M3. In multivariable analysis, these results stayed consistent for both the Söring (aOR, 0.74; 95 % CI, 0.37–1.47; *p* = 0.389) and the Sonopet group (aOR, 0.72; 95 % CI, 0.25–2.11; *p* = 0.553; Supplementary table 4).

### Secondary endpoint: in-hospital complications

Any complications until discharge occurred in a total of 308 patients (30.0 %); 118 (33.3 %), 120 (26.0 %), and 70 (32.9 %) in the CUSA, Söring, and Sonopet group, respectively (*p* = 0.049). The grading (*p* = 0.608) of complications was similar for all three UAs (Table 4).

**Table 4:** Occurrence and grading of in-hospital complications, according to the CDG scale

	CUSA	Söring	Sonopet	*p* value
Any complication				0.046
No	236 (66.7 %)	341 (74.0 %)	143 (67.1 %)	
Yes	118 (33.3 %)	120 (26.0 %)	70 (32.9 %)	
Complication grading (CDG)				0.608
1	55 (46.6 %)	57 (47.5 %)	32 (45.7 %)	
2	43 (36.4 %)	46 (38.3 %)	27 (38.6 %)	
3a	7 (5.9 %)	4 (3.3 %)	3 (4.3 %)	
3b	6 (5.1 %)	9 (7.5 %)	6 (8.6 %)	
4a	7 (5.9 %)	2 (1.7 %)	2 (2.9 %)	
5	– (0 %)	2 (1.7 %)	– (0 %)	
	*n* = *354* (*100* %)	*n* = *461* (*100* %)	*n* = *213* (*100* %)	

Data is presented in count (percent)

Italic entries were the count of the total cohort size

We specifically analyzed the occurrence of major complications, requiring invasive treatment under anesthesia (CDG 3b), transfer to the intensive care unit (CDG 4) or resulting in death (CDG 5). In uni- and multivariable analysis, patients in the Söring (aOR, 1.30; 95 % CI, 0.51–3.30; *p* = 0.583) and Sonopet group (aOR, 1.38; 95 % CI, 0.51–3.72; *p* = 0.524) were as likely to suffer from major complications (CDG 3b–5) as those in the CUSA group (Supplementary table 5).

The etiology of complications was comparable between the three groups (*p* = 0.849; Supplementary table 6).

For the scope of this project, complications resulting from direct surgical trauma were most interesting. Those occurred in 52 (14.7 %), 40 (8.7 %), and 26 (12.2 %) patients of the CUSA, Söring, and Sonopet group, respectively. In uni- and multivariable analysis, patients in both the Söring (aOR, 0.90; 95 % CI, 0.49–1.66; *p* = 0.740) and Sonopet group (aOR, 0.75, 95 % CI, 0.39–1.43; *p* = 0.374) were as likely as patients in the CUSA group to experience a traumatic surgical complication (Supplementary table 7). Patients with tumors in eloquent locations were 1.39× as likely as patients with tumors in non-eloquent locations to experience a traumatic surgical complication (95 % CI, 0.87–2.22; *p* = 0.168).

### Secondary endpoints: discharge location, LOS and LOH

Discharge locations were similar for all groups (*p* = 0.380) with a total of *n* = 699 (68.0 %) patients being discharged home, *n* = 11 (1.1 %) to a nursing home, *n* = 302 (29.4 %) to a rehabilitation clinic, and *n* = 16 (1.6 %) to another location (Supplementary table 8).

The mean LOS was 309.1 min (standard deviation SD, 133.1), 255.7 min (SD, 120.2), and 299.3 min (SD, 128.6) for the CUSA, Söring, and Sonopet group, respectively. In MANOVA, adjusted for baseline differences, there was a statistically significant difference between the three groups (*p* = 0.019; Supplementary figure 1).

The mean LOH for the CUSA, Söring, and Sonopet group was similar with 8.7 days (SD, 5.1), 8.1 days (SD, 5.0), and 8.9 days (SD., 5.2), respectively (*p* = 0.702; Supplementary figure 2).

### Secondary endpoint: EOR

The achieved EOR was GTR in *n* = 353 (34.3 %), STR in *n* = 372 (36.2 %), unclear in *n* = 261 (25.4 %), and no early postoperative imaging was available in *n* = 42 (4.1%). In uni- and multivariable analysis, surgeons using both the Söring (aOR, 1.14, 95 % CI, 0.84–1.56; *p* = 0.379) and Sonopet (aOR, 1.22; 95 % CI, 0.85–1.76; *p* = 0.387) were as likely as surgeons using the CUSA to achieve GTR of the tumor (Supplementary Table 9).

## Discussion

The aim of this study was to analyze the efficacy and safety of three commonly used UA types for the microsurgical resection of intracranial tumors by analyzing prospectively collected data on complications, clinical outcomes, and the EOR in a large, consecutive, and representative sample. This question is important, as a higher complication rate, a lower EOR, or a negative effect on patient outcome resulting from one specific UA type would have resulted in the necessity to reevaluate the safety of its use. No prior comparative analyses with regard to the resection of intracranial tumors were available.

The study cohort arrived at a reasonably large size of *n* = 1028 after excluding cases that otherwise would have lowered the accuracy and quality of the analysis. The sample size was sufficiently large to arrive at a power of more than 0.80 to detect a 5 % difference in the primary endpoint.

We analyzed baseline patient characteristics first, in an intention to identify possible confounders and sources of bias. Most variables such as age, smoking status, previous surgery, tumor size and—importantly—a patient’s functional status at admission (KPS, mRS, NIHSS) were evenly distributed across the study groups. However, there were significant differences in terms of sex, anesthesia risk (ASA class), tumor type, tumor location, procedural complexity (MCS; Table 1), and the surgeon’s level of experience.

These differences are most likely due to the surgeon’s selection of certain UA devices for patient- and disease-specific characteristics. A meningioma can be hard in consistency due to calcification and its resection requires a potent UA device that handles solid tissue safely. Since the Sonopet can also be used as a bone aspirator [11], our surgeons tend to choose this model for meningioma resections, which can be appreciated in this study: the Sonopet was used in *n* = 126 meningioma cases, whereas the CUSA and the Söring were used in *n* = 105 and *n* = 49 cases, respectively. The CUSA is particularly well-known for its precision [9, 17], the possibility of “tissue select” allowing to adjust the tissue-specific aspiration strength, and its broad choice of tips with different shapes and lengths. It is therefore preferred for procedures in the depth of the brain or skull base, as well as for the resection of tumors that are attached to blood vessels or cranial nerves, like schwannomas and skull base meningiomas. This can be seen in the distribution of cases labeled as “other” (including those entities) with *n* = 64, *n* = 28, and *n* = 27 cases in the CUSA, Söring, and Sonopet group, respectively. As Söring is the model in the series with the lowest costs for the disposable material, it is the most frequently used UA model for procedures where no particular difficulties are expected, such as gliomas (*n* = 247) and metastases (*n* = 137). Moreover, high-case complexity, measured using the MCS [8], was associated in particular with the use of CUSA (*n* = 73) rather than Sonopet (*n* = 51) or Söring (*n* = 28).

The KPS was used to measure morbidity at discharge and M3 follow-up. It was chosen for its close correlation to surgery-related outcomes and its predictive capacity for morbidity in intracranial tumor patients [18]. The current definition of morbidity had previously been used [12, 18]. As the prospective data registry [15] had complete data for the KPS at admission, discharge and M3 follow-up, we could eliminate additional selection bias due to missing data.

In the unadjusted analysis, the odds for discharge morbidity in the Söring group were higher than 1.0 (OR, 1.30, 95 % CI, 0.82–2.04; *p* = 0.252), likely an effect of the difference in histopathological tumor types. The Söring group included more than twice as many patients with metastases than the CUSA, while more patients with benign tumors were included in the CUSA group (Table 1). The risk estimate was corrected slightly downwards once adjusted for baseline differences in the multivariable model (aOR, 1.29, 95 % CI, 0.80–2.08; *p* = 0.299). As the point estimate is rather close to 1.0, the 95 % CIs appear narrow and the *p* value is well above 0.05, the use of Söring does not increase the likelihood for morbidity, in comparison to the CUSA. The odds for discharge morbidity in the Sonopet group were lower than 1.0 in the unadjusted model (OR, 0.81, 95 % CI, 0.44–1.50; *p* = 0.513), an effect most likely due to differences in case complexity and tumor type. Again, the results were comparable once adjusted for baseline group differences (aOR, 0.86; 95 % CI, 0.46–1.61; *p* = 0.645), with a point estimate close to 1.0 and narrow 95 % CIs. Consequently, the use of the Sonopet has neither a positive nor negative effect on the likelihood for discharge morbidity, compared to the CUSA (Table 2).

The two factors identified as independent predictors of discharge morbidity were increased in the ASA and MCS grade. Both results appear reasonable, as higher anesthesia risk and higher case complexity would be expected to have a negative impact on morbidity, suggesting that the data behind our analyses are valid. Additional sensitivity analyses showed robustness of the model.

Morbidity at M3 was analyzed to determine longer term effects of the UA types on patient outcome. The M3 time point was chosen, as for the outcome assessment at 1-year postoperative, the missing data burden was higher and the natural disease course would have confounded the relationship between UA type and functional outcome even more. In the multivariable analysis, the use of the Söring appeared to have no impact on M3 morbidity (aOR, 1.20; 95 % CI, 0.78–1.86; *p* = 0.415). However, we found a tendency for a lower likelihood of patients operated with the Stryker*®* Sonopet to experience morbidity at M3 (aOR, 0.53; 95 % CI, 0.26–1.08; *p* = 0.080). In order to evaluate, whether the lower risk arose from differences in the histopathological tumor type—despite the multivariable analysis—tumor-type-specific subgroup analyses were conducted. Those allowed us to exclude any bias resulting from the histopathological tumor type *à priori*. The analyses revealed no significant effect of the UA type on morbidity at M3 for *n* = 419 gliomas, *n* = 280 meningiomas, as well as *n* = 210 metastases (Supplementary tables 1–3). Despite the loss of power inherent to the smaller sample sizes, the subgroup analyses supported the notion that there was no significant effect of the UA type on morbidity at M3.

In this series, the in-hospital mortality was *n* = 2 patients (0.19 %), both of which occurred in the Söring group (*p* = 0.292). As mortality had increased at M3, logistic regression analysis of mortality was possible. In the adjusted model, both patients in the Söring (aOR, 0.74; *p* = 0.389) and Sonopet (aOR, 0.72; *p* = 0.553) group had a comparably low risk for M3 mortality. With *p* > 0.05, a significant effect of either UA type on M3 mortality can be excluded. This result is reasonable, as from clinical experience, we would not have expected any UA device to increase the mortality risk.

Both morbidity and mortality would likely result from a device-specific increase in surgical complications, which is why further analyses focused on those. Here, the rates of complications in the CUSA and Sonopet groups were comparable (about 33 %), while it was slightly lower in the Söring group (26.0 %; *p* = 0.049; Table 4). It should be acknowledged that the comparison of raw complication rates (Table 4) does not take into account the differences in case complexity. Complications were classified by the CDG scale, which indicates the type of treatment required to manage the complication. The CDG grading of complications was similar across the study groups (*p* = 0.608; Table 4).

As severe complications are usually more resource-intensive, we specifically analyzed “major complications,” represented by CDG 3b-5. The odds for patients in both the Söring and Sonopet groups to experience a major complication were similar to those of patients in the CUSA group (Supplementary table 5). Thus, significant harmful or protective effects from specific UA types on major complications could be excluded.

In theory, technical differences in the UA devices might best be evident from the specific analysis of injury to the brain. For example, devices that do not allow for fine adjustments in power or devices that are not easy to handle could theoretically translate into a higher rate of traumatic complications. We noticed that traumatic complications were among the leading causes of complications in all three groups (Supplementary table 6), and thus dedicated a further analysis to this specific complication etiology. Again, we found that—also after adjustment for baseline group differences—the odds for patients in both the Söring and Sonopet groups to experience a traumatic complication were similar to those in the CUSA group (Supplementary table 7).

A further analysis was dedicated to LOS, the reason being that technical (dis)advantages of any UA device for the resection of brain tumor tissue might translate into the procedural duration. The LOS appeared similar (mean of about 300 min) for procedures performed with the CUSA and Sonopet but was around 40 min shorter for those performed with the Söring (*p* = 0.019; Supplementary figure 1). As the Söring was typically used to resect soft and non-complex gliomas, the finding of shorter LOS may still be related to the marked differences in the histopathological tumor type and in case complexity—despite the statistical adjustment for group differences by a MANOVA model. The shorter LOS did not translate into better or worse clinical outcomes of patients in the Söring group.

We considered it important to analyze the EOR, since the quality of an UA device has the potential to influence it positively or negatively. In order to facilitate adjusted analyses, we focused on the likelihood to obtain GTR—usually the treatment goal aimed for in neurooncological surgery if the functional anatomy allows for it. Here, the odds for surgeons in both the Söring (aOR, 1.14; 95 % CI, 0.84–1.56; *p* = 0.379) and the Sonopet group (aOR, 1.22, 95 % CI, 0.85–1.76; *p* = 0.387) to achieve GTR of the tumor were closely to 1.0 in the adjusted models, indicating that both UA types are within the range of the CUSA to achieve GTR in patients (Supplementary table 9).

As opposed to the physician-rated and subjective KPS, both discharge location and LOH represent objective surrogate markers of outcome [1, 2, 10]. In our cohort, patients were discharged home in most cases (all study groups), followed by discharge to a rehabilitation clinic or other hospital. The distribution of discharge location was comparable for the three UA types (*p* = 0.380; Supplementary table 8). This finding, as well as the similar LOH across the study groups (*p* = 0.702), is in line with the previously illustrated similarity in KPS-based discharge morbidity across the UA types.

### Strengths and limitations

Distinct strengths of this study are its large cohort size (sufficiently powered for the main analysis) and very detailed, prospective data collection including a variety of variables representative of complications and different facets of the outcome.

Without doubt, the main drawback of this study is the strong selection bias, resulting from patient- and disease-specific factors that drove surgeons towards choosing a specific type of UA. Albeit, this finding being a very interesting result itself, it complicated the downstream analysis as it required us to employ mechanisms for statistical adjustment. Even despite adequate adjustment, it can never completely control the important between-group heterogeneities. In theory, a trial where the type of UA would be randomly assigned to patients—regardless of their tumor type and case complexity—would be optimal to address our research question. Such a trial, however, might experience resistance from surgeons, who like to select the surgical tools they consider optimal for the individual case. It might even affect patient safety in a negative way by allocating an UA model less suitable for the surgeon to the individual case, therefore be unethical. In fact, our surgeons may have intentionally selected the optimal UA type for the individual case, hereby obscuring differences in the performance of the UA types and their effect on the studied outcomes. However, as the detected differences in outcome in our present analysis were minor, a trial would need to study a large sample size and be expensive. It is unlikely that such a trial will ever be conducted and therefore cross-sectional observational studies may be the best evidence we can currently generate.

The inclusion of many different outcome variables can be considered a strength of this work. On the one hand, if an analysis studying a relationship from many different angels arrives at the same conclusions—regardless of the outcome measure—one can be confident that the results represent meaningful, true findings. In our study, almost all findings uniformly pointed towards the same effect: similarity in clinical outcome, complications, and EOR for all three UA types. On the other hand, employing various outcome variables inevitably resulted in a high number of statistical tests and results, increasing the likelihood of type-I errors. We would have applied Bonferroni post-hoc adjustment to critically evaluate any finding suggesting a distinct difference between study groups. As the results indicated gross similarity, this was not considered necessary.

There has been no prior work published so far on the comparative safety profiles of UAs in neurosurgery. Therefore, this research currently stands alone and cannot be compared to previous literature. Since UAs are commonly used nowadays and many models from different companies are available, we encourage other groups to provide more high-quality data on this question. Further studies on the comparative safety profiles of UA types should be conducted, encompassing intracranial, but possibly also intraspinal tumor patients. Those studies are valuable to help surgeons select appropriate tools for efficient and safe surgical procedures, with an ultimately positive impact on patient well-being.

## Conclusions

Neurosurgeons select UA types with regard to certain case-specific characteristics. The safety profiles of three commonly used UA types appear mostly similar.

## Electronic supplementary material

ESM 1 (DOCX 331 kb)

ESm 2 (DOCX 99 kb)

ESM 3 (DOCX 18 kb)

ESM 4 (DOCX 17 kb)

ESM 5 (DOCX 17 kb)

ESM 6 (DOCX 18 kb)

ESM 7 (DOCX 18 kb)

ESM 8 (DOCX 16 kb)

ESM 9 (DOCX 18 kb)

ESM 10 (DOCX 16 kb)

ESM 11 (DOCX 18 kb)

## Abbreviations and acronyms

ANOVA: Univariable analysis of variance
aOR: Adjusted odds ratio
ASA: American Society of Anesthesiology
CDG: Clavien-Dindo grading scale
CI: Confidence interval
EOR: Extend of resection
GTR: Gross total resection
KPS: Karnofsky Performance Scale
LOH: Length of hospitalization
LOS: Length of surgery
MANOVA: Multivariable analysis of variance
MCS: Milan Complexity Score
MRI: Magnetic resonance imaging
mRS: Modified Rankin scale
M3: Three months postoperative
NIHSS: National Institute of Health stroke scale
SD: Standard deviation
STR: Subtotal resection
UA: Ultrasonic aspiration
USZ: University Hospital Zurich
