# Supplementary material for: Ultrasonic aspiration in neurosurgery: comparative analysis of complications and outcome for three commonly used models
Source: Acta Neurochir (Wien). 2019 Aug 3;161(10):2073–82. doi: 10.1007/s00701-019-04021-0 (PMC6739453; doi:10.1007/s00701-019-04021-0)
Supplement: Supplementary file 2 — (DOCX 99 kb) [file 701_2019_4021_MOESM2_ESM.docx]

**Supplementary figure 2: Length of hospitalization.**


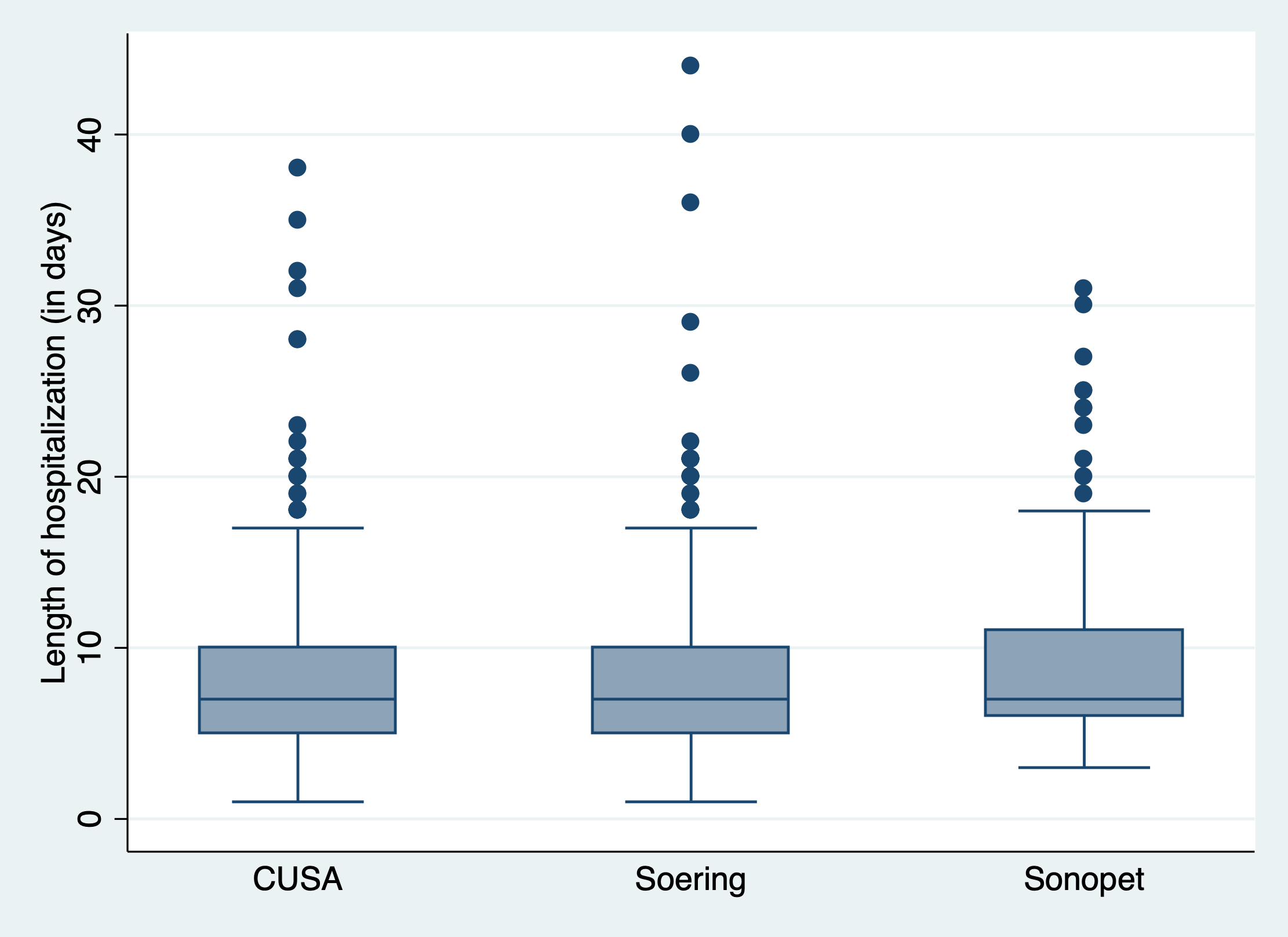


The figures display the median with 25^th^ – 75^th^ percentile (box), the upper and lower adjacent values (whiskers) and outliers (dots).

**Ultrasonic aspiration in neurosurgery: comparative analysis of complications and outcome for three commonly used models**

Stephanie Henzi^1,2^, MMed; Niklaus Krayenbühl^1,2^, MD; Oliver Bozinov^1,2^, MD; Luca Regli, MD; Martin N. Stienen^1,2^, MD/FEBNS

^1^ Department of Neurosurgery, University Hospital Zurich, Zurich, Switzerland

^2^ Clinical Neuroscience Center, University of Zurich, Zurich, Switzerland

**Corresponding author:**

Martin N. Stienen, MD

Fellow of the European Board of Neurological Surgeons (FEBNS)

University Hospital Zurich

Clinical Neuroscience Center

University of Zurich

Frauenklinikstrasse 10

8091 Zurich, Switzerland

Tel: +41-(0)44-255-1111

Email: [mnstienen@gmail.com](mailto:mnstienen@gmail.com)
