# Supplementary material for: Ultrasonic aspiration in neurosurgery: comparative analysis of complications and outcome for three commonly used models
Source: Acta Neurochir (Wien). 2019 Aug 3;161(10):2073–82. doi: 10.1007/s00701-019-04021-0 (PMC6739453; doi:10.1007/s00701-019-04021-0)
Supplement: Supplementary file 4 — (DOCX 17 kb) [file 701_2019_4021_MOESM4_ESM.docx]

**Supplementary table 2: Relationship between UA type and morbidity at M3 follow-up of meningioma patients.**

| **M3 morbidity of**  **meningioma patients** | **Univariate analysis** | | | **Multivariate analysis** | | |
| --- | --- | --- | --- | --- | --- | --- |
|  | **OR** | **95% CI** | **p-value** | **OR** | **95% CI** | **p-value** |
| UA type*  Söring  Sonopet | 0.34  0.27 | 0.04 – 2.93  0.05 – 1.35 | 0.329  0.110 | 0.87  0.25 | 0.08 – 8.92  0.05 – 1.31 | 0.903  0.100 |
| Female sex |  |  |  | 0.88 | 0.16 – 4.84 | 0.878 |
| ASA grade  (per 1-step increase) |  |  |  | 1.25 | 0.38 – 4.07 | 0.714 |
| MCS grade  (per increase in category) |  |  |  | 5.10 | 1.42 – 17.94 | 0.012 |
| Level of experience |  |  |  | 1.91 | 0.38 – 9.61 | 0.430 |

Uni- and multivariate logistic regression analysis estimating the relationship between UA type and morbidity at time of M3 follow-up in n=280 meningioma patients. The multivariate analysis is adjusted for baseline differences in sex, ASA grading scale, the case complexity (MCS) and level of experience. *The analysis compares the results of each listed UA type with the CUSA ultrasonic aspirator.

**Ultrasonic aspiration in neurosurgery: comparative analysis of complications and outcome for three commonly used models**

Stephanie Henzi^1,2^, MMed; Niklaus Krayenbühl^1,2^, MD; Oliver Bozinov^1,2^, MD; Luca Regli, MD; Martin N. Stienen^1,2^, MD/FEBNS

^1^ Department of Neurosurgery, University Hospital Zurich, Zurich, Switzerland

^2^ Clinical Neuroscience Center, University of Zurich, Zurich, Switzerland

**Corresponding author:**

Martin N. Stienen, MD

Fellow of the European Board of Neurological Surgeons (FEBNS)

University Hospital Zurich

Clinical Neuroscience Center

University of Zurich

Frauenklinikstrasse 10

8091 Zurich, Switzerland

Tel: +41-(0)44-255-1111

Email: [mnstienen@gmail.com](mailto:mnstienen@gmail.com)
