# Supplementary material for: Ultrasonic aspiration in neurosurgery: comparative analysis of complications and outcome for three commonly used models
Source: Acta Neurochir (Wien). 2019 Aug 3;161(10):2073–82. doi: 10.1007/s00701-019-04021-0 (PMC6739453; doi:10.1007/s00701-019-04021-0)
Supplement: Supplementary file 5 — (DOCX 17 kb) [file 701_2019_4021_MOESM5_ESM.docx]

**Supplementary table 3: Relationship between UA type and morbidity at M3 follow-up of metastasis patients.**

| **M3 morbidity of**  **metastasis patients** | **Univariate analysis** | | | **Multivariate analysis** | | |
| --- | --- | --- | --- | --- | --- | --- |
|  | **OR** | **95% CI** | **p-value** | **OR** | **95% CI** | **p-value** |
| UA type*  Söring  Sonopet | 0.82  0.58 | 0.40 – 1.70  0.15 – 2.33 | 0.596  0.447 | 0.76  0.57 | 0.35 – 1.61  0.14 – 2.32 | 0.469  0.432 |
| Female sex |  |  |  | 1.80 | 0.93 – 3.50 | 0.084 |
| ASA grade  (per 1-step increase) |  |  |  | 1.44 | 0.84 – 2.46 | 0.190 |
| MCS grade  (per increase in category) |  |  |  | 0.96 | 0.51 – 1.80 | 0.887 |
| Level of experience |  |  |  | 0.86 | 0.53 – 1.40 | 0.551 |

Uni- and multivariate logistic regression analysis estimating the relationship between UA type and morbidity at time of M3 follow-up in n=210 metastasis patients. The multivariate analysis is adjusted for baseline differences in sex, ASA grading scale, the case complexity (MCS) and level of experience. *The analysis compares the results of each listed UA type with the CUSA ultrasonic aspirator.

**Ultrasonic aspiration in neurosurgery: comparative analysis of complications and outcome for three commonly used models**

Stephanie Henzi^1,2^, MMed; Niklaus Krayenbühl^1,2^, MD; Oliver Bozinov^1,2^, MD; Luca Regli, MD; Martin N. Stienen^1,2^, MD/FEBNS

^1^ Department of Neurosurgery, University Hospital Zurich, Zurich, Switzerland

^2^ Clinical Neuroscience Center, University of Zurich, Zurich, Switzerland

**Corresponding author:**

Martin N. Stienen, MD

Fellow of the European Board of Neurological Surgeons (FEBNS)

University Hospital Zurich

Clinical Neuroscience Center

University of Zurich

Frauenklinikstrasse 10

8091 Zurich, Switzerland

Tel: +41-(0)44-255-1111

Email: [mnstienen@gmail.com](mailto:mnstienen@gmail.com)
