# Supplementary material for: Ultrasonic aspiration in neurosurgery: comparative analysis of complications and outcome for three commonly used models
Source: Acta Neurochir (Wien). 2019 Aug 3;161(10):2073–82. doi: 10.1007/s00701-019-04021-0 (PMC6739453; doi:10.1007/s00701-019-04021-0)
Supplement: Supplementary file 7 — (DOCX 18 kb) [file 701_2019_4021_MOESM7_ESM.docx]

**Supplementary table 5: Relationship between UA type and occurrence of a major complication (CDG grade 3b–5).**

| **Major complications** | **Univariate analysis** | | | **Multivariate analysis** | | |
| --- | --- | --- | --- | --- | --- | --- |
|  | **OR** | **95% CI** | **p-value** | **OR** | **95% CI** | **p-value** |
| UA type*  Söring  Sonopet | 0.98  1.04 | 0.43 – 2.21  0.41 – 2.65 | 0.964  0.931 | 1.30  1.38 | 0.51 – 3.30  0.51 – 3.72 | 0.583  0.524 |
| Female sex |  |  |  | 2.73 | 1.23 – 6.02 | 0.013 |
| ASA grade  (per 1-step increase) |  |  |  | 1.96 | 1.11 – 3.47 | 0.018 |
| Tumor type |  |  |  | 1.43 | 1.01 – 2.01 | 0.041 |
| Extraaxial tumor location |  |  |  | 1.56 | 0.74 – 3.30 | 0.242 |
| MCS grade  (per increase in category) |  |  |  | 1.33 | 0.78 – 2.28 | 0.290 |
| Level of experience |  |  |  | 0.98 | 0.53 – 1.81 | 0.952 |

Uni- and multivariate logistic regression analysis estimating the relationship between UA type and major complication. The multivariate analysis is adjusted for baseline differences in sex, ASA grading scale, type of tumor, location of tumor, the case complexity (MCS) and level of experience. *The analysis compares the results of each listed UA type with the CUSA ultrasonic aspirator.

**Ultrasonic aspiration in neurosurgery: comparative analysis of complications and outcome for three commonly used models**

Stephanie Henzi^1,2^, MMed; Niklaus Krayenbühl^1,2^, MD; Oliver Bozinov^1,2^, MD; Luca Regli, MD; Martin N. Stienen^1,2^, MD/FEBNS

^1^ Department of Neurosurgery, University Hospital Zurich, Zurich, Switzerland

^2^ Clinical Neuroscience Center, University of Zurich, Zurich, Switzerland

**Corresponding author:**

Martin N. Stienen, MD

Fellow of the European Board of Neurological Surgeons (FEBNS)

University Hospital Zurich

Clinical Neuroscience Center

University of Zurich

Frauenklinikstrasse 10

8091 Zurich, Switzerland

Tel: +41-(0)44-255-1111

Email: [mnstienen@gmail.com](mailto:mnstienen@gmail.com)
