# Supplementary material for: Ultrasonic aspiration in neurosurgery: comparative analysis of complications and outcome for three commonly used models
Source: Acta Neurochir (Wien). 2019 Aug 3;161(10):2073–82. doi: 10.1007/s00701-019-04021-0 (PMC6739453; doi:10.1007/s00701-019-04021-0)
Supplement: Supplementary file 8 — (DOCX 16 kb) [file 701_2019_4021_MOESM8_ESM.docx]

**Supplementary table 6: Etiology of in-hospital complications.**

|  | **CUSA** | **Söring** | **Sonopet** | **p-value** |
| --- | --- | --- | --- | --- |
| CSF-related  Epilepsy  General medicine  Hemorrhagic  Ischemic  Septic  Traumatic  Other | 6 (5.1%)  10 (8.5%)  26 (22.0%)  4 (3.4%)  14 (11.9%)  1 (0.9%)  52 (44.1%)  5 (4.2%) | 3 (2.5%)  11 (9.2%)  33 (27.5%)  9 (7.5%)  14 (11.7%)  3 (2.5%)  40 (33.3%)  7 (5.8%) | 3 (4.3%)  7 (10.0%)  19 (27.1%)  6 (8.6%)  6 (8.6%)  1 (1.4%)  26 (37.1%)  2 (2.9%) | 0.849 |
|  | **n=118 (100%)** | **n=120 (100%)** | **n=70 (100%)** |  |

Data is presented in count (percent).

**Ultrasonic aspiration in neurosurgery: comparative analysis of complications and outcome for three commonly used models**

Stephanie Henzi^1,2^, MMed; Niklaus Krayenbühl^1,2^, MD; Oliver Bozinov^1,2^, MD; Luca Regli, MD; Martin N. Stienen^1,2^, MD/FEBNS

^1^ Department of Neurosurgery, University Hospital Zurich, Zurich, Switzerland

^2^ Clinical Neuroscience Center, University of Zurich, Zurich, Switzerland

**Corresponding author:**

Martin N. Stienen, MD

Fellow of the European Board of Neurological Surgeons (FEBNS)

University Hospital Zurich

Clinical Neuroscience Center

University of Zurich

Frauenklinikstrasse 10

8091 Zurich, Switzerland

Tel: +41-(0)44-255-1111

Email: [mnstienen@gmail.com](mailto:mnstienen@gmail.com)
