# Supplementary material for: Ultrasonic aspiration in neurosurgery: comparative analysis of complications and outcome for three commonly used models
Source: Acta Neurochir (Wien). 2019 Aug 3;161(10):2073–82. doi: 10.1007/s00701-019-04021-0 (PMC6739453; doi:10.1007/s00701-019-04021-0)
Supplement: Supplementary file 9 — (DOCX 18 kb) [file 701_2019_4021_MOESM9_ESM.docx]

**Supplementary table 7: Relationship between UA type and a traumatic surgical complication.**

| **Traumatic surgical complication** | **Univariate analysis** | | | **Multivariate analysis** | | |
| --- | --- | --- | --- | --- | --- | --- |
|  | **OR** | **95% CI** | **p-value** | **OR** | **95% CI** | **p-value** |
| UA type*  Söring  Sonopet | 0.63  0.75 | 0.37 – 1.07  0.41 – 1.37 | 0.090  0.352 | 0.90  0.75 | 0.49 – 1.66  0.39 – 1.43 | 0.740  0.374 |
| Female sex |  |  |  | 0.76 | 0.46 – 1.25 | 0.273 |
| ASA grade  (per 1-step increase) |  |  |  | 0.55 | 0.37 – 0.80 | 0.002 |
| Tumor type |  |  |  | 1.12 | 0.89 – 1.42 | 0.331 |
| Extraaxial tumor location |  |  |  | 0.63 | 0.38 – 1.06 | 0.083 |
| MCS grade  (per increase in category) |  |  |  | 1.30 | 0.91 – 1.86 | 0.157 |
| Level of experience |  |  |  | 1.38 | 0.93 – 2.06 | 0.112 |

Uni- and multivariate logistic regression analysis estimating the relationship between UA type and a traumatic surgical complication. The multivariate analysis is adjusted for baseline differences in sex, ASA grading scale, type of tumor, location of tumor, the case complexity (MCS) and level of experience. *The analysis compares the results of each listed UA type with the CUSA ultrasonic aspirator.

**Ultrasonic aspiration in neurosurgery: comparative analysis of complications and outcome for three commonly used models**

Stephanie Henzi^1,2^, MMed; Niklaus Krayenbühl^1,2^, MD; Oliver Bozinov^1,2^, MD; Luca Regli, MD; Martin N. Stienen^1,2^, MD/FEBNS

^1^ Department of Neurosurgery, University Hospital Zurich, Zurich, Switzerland

^2^ Clinical Neuroscience Center, University of Zurich, Zurich, Switzerland

**Corresponding author:**

Martin N. Stienen, MD

Fellow of the European Board of Neurological Surgeons (FEBNS)

University Hospital Zurich

Clinical Neuroscience Center

University of Zurich

Frauenklinikstrasse 10

8091 Zurich, Switzerland

Tel: +41-(0)44-255-1111

Email: [mnstienen@gmail.com](mailto:mnstienen@gmail.com)
