# Supplementary material for: Ultrasonic aspiration in neurosurgery: comparative analysis of complications and outcome for three commonly used models
Source: Acta Neurochir (Wien). 2019 Aug 3;161(10):2073–82. doi: 10.1007/s00701-019-04021-0 (PMC6739453; doi:10.1007/s00701-019-04021-0)
Supplement: Supplementary file 10 — (DOCX 16 kb) [file 701_2019_4021_MOESM10_ESM.docx]

**Supplementary table 8: Discharge location.**

|  | **CUSA** | **Soering** | **Sonopet** | **p-value** |
| --- | --- | --- | --- | --- |
| Home  Nursing home  Rehabilitation / Clinic  Other | 253 (71.5%)  5 (1.4%)  91 (25.7%)  5 (1.4%) | 312 (67.7%)  3 (0.7%)  138 (29.9%)  8 (1.7%) | 134 (62.9%)  3 (1.4%)  73 (34.3%)  3 (1.4%) | 0.380 |
|  | **n=354 (100%)** | **n=461 (100%)** | **n=213 (100%)** |  |

Data is presented in count (percentage).

**Ultrasonic aspiration in neurosurgery: comparative analysis of complications and outcome for three commonly used models**

Stephanie Henzi^1,2^, MMed; Niklaus Krayenbühl^1,2^, MD; Oliver Bozinov^1,2^, MD; Luca Regli, MD; Martin N. Stienen^1,2^, MD/FEBNS

^1^ Department of Neurosurgery, University Hospital Zurich, Zurich, Switzerland

^2^ Clinical Neuroscience Center, University of Zurich, Zurich, Switzerland

**Corresponding author:**

Martin N. Stienen, MD

Fellow of the European Board of Neurological Surgeons (FEBNS)

University Hospital Zurich

Clinical Neuroscience Center

University of Zurich

Frauenklinikstrasse 10

8091 Zurich, Switzerland

Tel: +41-(0)44-255-1111

Email: [mnstienen@gmail.com](mailto:mnstienen@gmail.com)
